# Effects of Vitamin-E treatment on CatSper genes expression and sperm quality in the testis of the aging mouse

**Published:** 2013-12

**Authors:** Shabnam Mohammadi, Mehdi Jalali, Mohammad Reza Nikravesh, Alireza Fazel, Alireza Ebrahimzadeh, Mehran Gholamin, Mojtaba Sankian

**Affiliations:** 1 *Department of Anatomy and Cell Biology, School of Medicine, Mashhad University of Medical Sciences, Mashhad, Iran.*; 2 *Division of Human Genetics, Immunology Research Center, Avicenna Research Institute, Mashhad University of Medical Sciences, Mashhad, Iran.*; 3 *Bou-ali Research Institute, Immunology Research Center, Mashhad University of Medical Sciences, Mashhad, Iran.*

**Keywords:** *CatSper*, *Sperm*, *Testis*, *Mice*, *Aging*, *Vitamin E*

## Abstract

**Background:** CatSper genes are a novel family of four sperm-specific calcium channels, which indicate testis-specific expression patterns. Despite the crucial role of CatSper genes in the male reproduction, very little is known about the factors that regulate their expression.

**Objective: **The objective of this study was to investigate the effects of vitamin E treatment on the expression of CatSper 1 and CatSper 2 genes as well as sperm quality in the aged male mice.

**Materials and Methods:** Twenty four 11-12 months old aged male mice and twenty four 2-3-months old young male mice were randomly divided into four groups. Control groups received no injection. The experimental groups of male mice were received intraperitoneal injection of 106 mg/kg vitamin E daily for 35 days. Left testis and cauda epididymides from each mouse were collected on the days 21, 28 and 35 following vitamin E treatment and were used for Real-Time PCR and immunohistochemistry. Also, sperm analysis was performed according to the WHO guidelines given for human sperm examination. Data were analyzed using SPSS software.

**Results:** Administration of vitamin E improved sperm parameters in the aged as well as young adult male mice. In addition, the expression of CatSper genes increased following vitamin E treatment. Also, intensity of signal for CatSper1 and CatSper2 increased in the head and middle piece of sperm in experimental group as compared to those of control ones.

**Conclusion:** The vitamin E treatment significantly improved the sperm quality, especially in terms of sperm motility, count and morphology rate. Furthermore, CatSper genes expression could be up-regulated by the vitamin E treatment.

This article extracted from Ph.D. thesis. (Shabnam Mohammadi)

## Introduction

During mammalian fertilization, calcium ions are important for sperm capacitation, motility, acrosome reaction, and penetration into the oocyte (1). Several ca+^2^-permeable channels have been identified in sperm cells, including high voltage-gated calcium channels, transient receptor potential channels, and cyclic nucleotide-gated channels (2). CatSpers are unique channels having a six transmembrane- spanning repeat and a single pore resembling that of the voltage-gated Ca^+2-^ permeant channels. These channels are exclusively expressed in the testis and highly conserved in the mouse and human (2, 3). 

The CatSpers contains four alpha subunits (CatSper 1-4) form a Ca^+2^ selective pore, and two additional auxiliary subunits, CatSper β and CatSper ɣ (2-5). CatSper1 and CatSper2 are critical to sperm motility and male fertility; whereas CatSper3 and CatSper3 play an important role in the sperm hyperactivated mobility and acrosome reaction (2, 6-10). Aging causes many of detrimental alternations in the male reproductive tract, such as testicular damage, decrease in sperm parameters and gene expression. Based on the free radical theory of aging, excessive production of free radicals causes the process of aging (11). 

It has been hypothesized that the balance between oxidant and antioxidant spices is one of the key regulators of the aging process. The semen contains a number of antioxidants such as glutathione peroxidase, superoxide dismutase, vitamin E, vitamin C, selenium and carnitine that protect spermatozoa against free radicals (12). Vitamin E is a lipid-soluble antioxidant that protects sperm cells from lipid peroxidation and oxidative stress (13). Vitamin E supplementation hasincreased the number and motility of sperm in animals (14, 15). 

Suleiman *et al* have reported that vitamin E administration improved sperm motility and declined Malondialdehyde (MDA) level in men with asteno or oligoasthenospermia (16). Conversely, dietary deficiency of vitamin E leads to deleterious changes on the male reproductive tract, such as histological alternations in seminiferous tubules and degenerative spermatogonium (17). Our previous study indicated that the supplementation with selenium in the aged mice could up-regulate the expression of CatSper genes, and improved sperm quality in the aged mice (18). 

Hence, the aim of the present study was to evaluate the effects of vitamin E, synergist of the selenium on the expression of CatSper genes and sperm quality in 11-12 months old aged and 2-3 months old young male mice.

## Materials and methods

**Chemicals**

Vitamin E (ɑ-tocopherol acetate) was manufactured by Sigma Corporation, USA. 

**Animals**

Male BALB/c mice, varying in age (the young group: n=48, 2-3 months old; the aged group: n=48, 11-12 months old) were purchased from the Experimental Animal Center of the Mashhad University of Medical Sciences, Mashhad, Iran. The mice were fed a standard chow and water ad libitum, and exposed to a 12-hour light/dark cycle, at a temperature of 22^o^C. All the experimental protocols were approved by the Ethical Committee of Mashhad University of Medical Science. 

**Study design**

In this experimental study, mice were randomly divided into four groups of twelve animals each (n=12): the Aged control mice (Control 1); the Aged mice receiving vitamin E treatment (Experimental 1); the Young control mice (Control 2) and the Young mice receiving vitamin E treatment (Experimental 2). The control groups received no injection. The experimental groups were administered, intraperitoneally, 106 mg/kg all-rac-a tocopheryl acetate for 35 days (11). The mice were dissected to collect the left testis and cauda epididymis from each group on the days 21, 28 and 35 after injection. Testis was stored at -80ºC until further analysis and sperm cells from the epididymis were used for sperm parameters.

**Sperm quality analysis**

Sperm analysis was performed according to WHO protocol given for human sperm examination (19). 

**Sperm Motility**

The left cauda epididymis was placed in 1 ml of phosphate buffer saline solution. Cauda was minced with scalpels and incubated in a 5% CO_2_ incubator for 15 min. One drop of sperm suspension was placed on a Neubauer chamber, covered by a 22×22 mm cover slip, and the percentage of motile sperm was evaluated under a light microscope at ×400 magnifications. 

**Sperm Count**

Sperms acquired from the epididymis were released into 1 ml of phosphate buffer saline. After 15 min incubation in a 5% CO_2_ incubator, sperm count was determined using a Neubaur hemocytometer under a light microscope (Olympus BH2). The sperm count was expressed as ×10^6^/mL. 

**Sperm Morphology**

One hundred sperm from different fields were counted for each animal to determine the morphological abnormalities. 

**Sperm Viability**

Two volume of Eosin-B was mixed with one volume of sperm sample. Dead sperm cells were stained red while live sperm cells were unstained. One hundred sperm cells were counted for each sperm sample and were expressed as the percent of viable sperm. 

**RNA isolation**

Total RNA was isolated from testis using the RNX plus solution (Cinnagen, Iran) according to the manufacturer's instructions. Briefly, the tissue was homogenized in 1 ml of RNX solution and incubated at room temperature for 5 min. Then, 200 µl of chloroform was added to the tube and centrifuged at 12000 g, for 15 min at 4^o^C. The upper phase containing RNA was transferred into a tube and an equal volume of isopropanol was added. The mixture was then centrifuged at 12000 g, at 4^o^C, for 10 min. The pellet was washed with 75% ethanol and resuspended in 50 µl of Diethylpyrocarbonate (DEPC) treated water. The RNA quantity and integrity was evaluated by the absorbance ratio A260/A280 nm and1% agarose gel electrophoresis, respectively.

**Reverse transcription**

cDNA Synthesis Kit Revert Aid were purchased from Fermentas Corporation (Germany). To synthesize cDNA, 1 µg of total RNA was reverse transcribed with 5X Reaction Buffer, 20 U/μl Ribolock RNase inhibitor, 10 mM dNTP, 200 U/μl MMLV reverse transcriptase, and oligo (dt)18 primer in a 20 μl reaction. The mixture was incubated at 42^o^C for 60 min, and then the enzyme was inactivated at 70^o^C for 5 min. 

**Real time**

Real-time PCR was carried out using SYBER Green fluorescence dye (Fermentas Corporation, Germany) and a M3000P Real-Time instrument (Stratagene, CA, USA). All reactions were run in duplicate for CatSper 1, CatSper 2 and β-Actin. The Primer sets were described elsewhere (20). Standard curves were constructed by using the logarithmic dilution series of complementary DNA from the high quality sample. Reaction mixtures was included 2 μl cDNA, 12.5 μl SYBER Green PCR Master Mix (Fermentase Corporation, Germany), 0.6 μl reverse primer, 0.6 μl forward primer, and 9.3 μl of water. Reaction conditions were as follows: an initial denaturation step (95^o^C for 10 min) followed by 40 cycles of a three-phase PCR (denaturation at 95^o^C for 25 s; annealing at 60^o^C for 30 s, and extension at 72^o^C for 30 s). β-actin was used as an internal control to normalize the Real time -PCR reaction. Relative gene expressions were calculated by using the Pfaffl method and β-actin as reference gene (21).

**Immunohistochemistry study**

Immunohistochemistry was carried out with the sperm cells that were collected from the mouse cauda epididymidis. A 10 μL of sperm sample was pipetted onto a poly-L-lysine-coated slide, air dried and fixed in cold methanol. Antigen retrieval was carried out by heating the slides in Tris/EDTA pH 9.0 at 95^o^C. The slides were blocked in 3% BSA for 2 hours at room temperature. For blocking endogenous peroxides activity, the slides were incubated in 3% H_2_O_2_^-^ methanol for 20 min. 

After three washes in PBS, slides were incubated in primary antibodies (mouse anti-goat CatSper antibody dilution 1:100) overnight at 4^o^C. The slides were washed three times with PBS, and incubated with secondary antibody (1:100 dilution; HRP-conjugated donkey anti-goat IgG) for 2 hours at room temperature. After 10 min washes in PBS, a 0.03% solution of diaminobenzidine was applied until brown color developed. Following wash, the samples were treated in xylene and dehydrated in a graded ethanol series 70-100%. Samples were mounted with Entellan (Merk, Germany). Slides incubated in PBS without primary antibody were used as negative controls. Microscopic images were obtained on an Olympus BX51 light microscope. After taking photomicrographs, the sperm staining patterns were determined semiquantitatively with the use of a 5-point scale: weak, +; moderate, ++; strong, +++; highly strong, ++++ (22). 

**Statistical analysis**

All statistical analysis was undertaken sing the statistical package for social sciences (SPSS v.21) software. Data were analyzed using ANOVA followed by Duncan post hoc test. P-value<0.05 was considered statistically significant. 

## Results

**Effects of Vitamin E treatment on sperm parameters in the aged and the young male mice**

The results of sperm characteristics for all groups are summarized in Table I. The analyses of variance demonstrated a significant difference between the aged control and the young control group (p<0.001). Treatment of mice with vitamin E caused a remarkable increase in the sperm count on the day 21 and 28 of the vitamin administration. 

Our findings revealed an increase in sperm motility of mice treated with vitamin E at the end of the experimental period, though this increase was not statistically significant in the experimental group 2 (p>0.05). In addition, the percentage of normal sperm morphology was increased in mice that received 106 mg/kg vitamin E. The types of sperm abnormalities noted were coiled tail, cooked tail, and vacuolated head. The percentage of live sperm cells was not affected by the vitamin treatment in both aged and young mice (p>0.05). 

**Effects of Vitamin E treatment on CatSper genes expression in the aged and the young male mice**

**Figure1:**
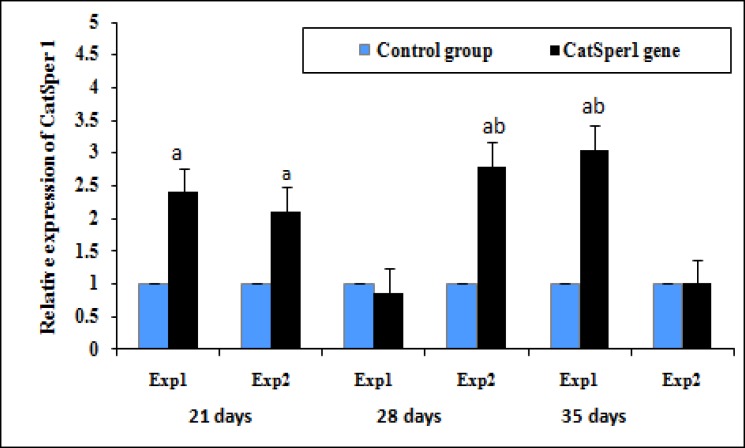
Effect of vitamin E treatment on mRNA levels of CatSper 1 in the aged and young mice Values expressed as means+SD, n=12 for each group. Exp: experimental.

^a^ significant differences (p<0.05) between control and experimental group

^b^ significant differences (p<0.05) between experimental group 1 and experimental group 2 within one time-point

**Figure 2:**
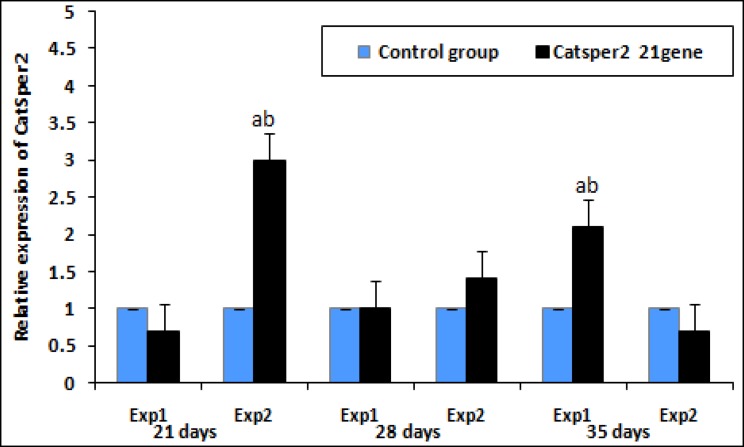
Effect of vitamin E treatment on mRNA levels of CatSper 2 in the aged and young mice

The results of relative expression of CatSper genes are shown in Figure 1-2. We observed different pattern in the expression of CatSper genes in the testis of the aged and young mice. The relative expression of CatSper 1 in the experimental group 1 was 2.40-fold greater than in control group 1 (2.40±0.66 vs. control group, p<0.01). The expression level of CatSper 1 was decreased at 28 days (0.85±0.24 vs. control group), and then the expression increased distinctly at 35 days (3.05-fold). At 21 days, the relative expression of CatSper 1 gene was 2.10-fold greater in experimental group 2 than those in the control group2 (p<0.001), reached a peak at 28 days, and then declined to its lowest level at 35 days (Figure 1).

The level of CatSper expression in experimental group1 decreased at 21 days at 0.70±0.24 fold change (p=0.001), compared to control, and then gradually increased at 28 and 35 days of vitamin treatment. The relative expression of CatSper 2 in the experimental group 2 increased at 21 days. The expression level of CatSper 2 declined significantly at 1.40-fold change at 28 days (1.40±0.41 vs. control group 2, p<0.01) and continued to decrease at 35 days (Figure 2).

**Figure 3:**
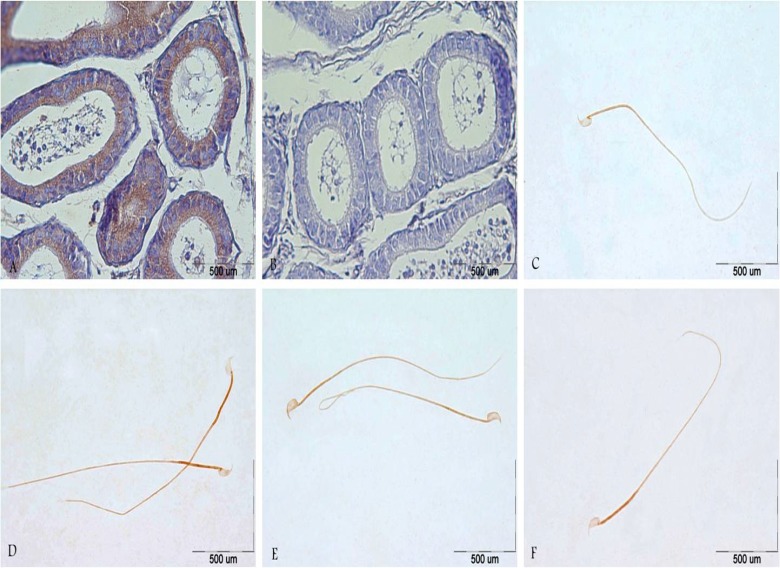
**(a)** Strong immunoreactivity of CatSper 2 was detected in the lumen of epididymis in the young control group. **(b)** Epididymis section of young control group without primary antibody, CatSper1, no reaction was observed. **(c,d)** localization of CatSper 1 protein in the head and sperm tail of the control group 1 **(c)** and experimental group 1 **(d). (e, f)** localization of CatSper 1 protein in the head and sperm tail of the control group 2 **(e)** and experimental group 2 **(f).** In all slides, immunoreactions visualization was done with DAB and counterstained with haematoxylin. Scale bars represent 500 µm.

**Figure 4:**
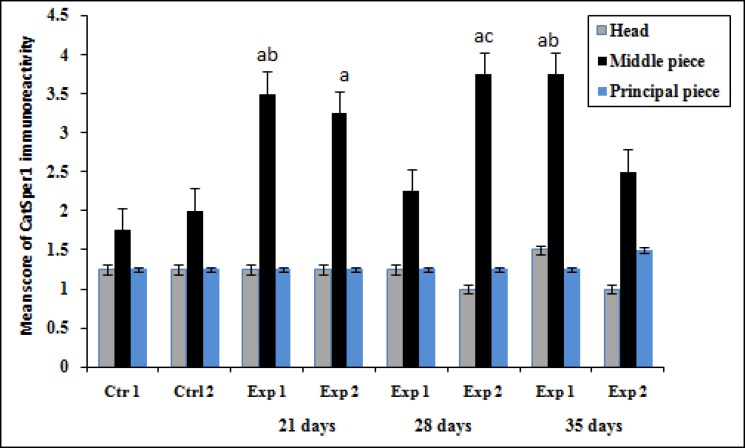
Effect of vitamin E treatment on protein levels of CatSper 1 in the aged and young mice Values expressed as means+SD, n=12 for each group. Exp: experimental, Ctrl: control

^a^ significant differences (p<0.05) between control 1 and experimental 1 group.

^b^ significant differences (p<0.05) between experimental group 1 on the days 21, 35 and the day 28.

^c^ significant differences (p<0.05) between experimental group 2 on the days 28 and 35.

**Figure 5:**
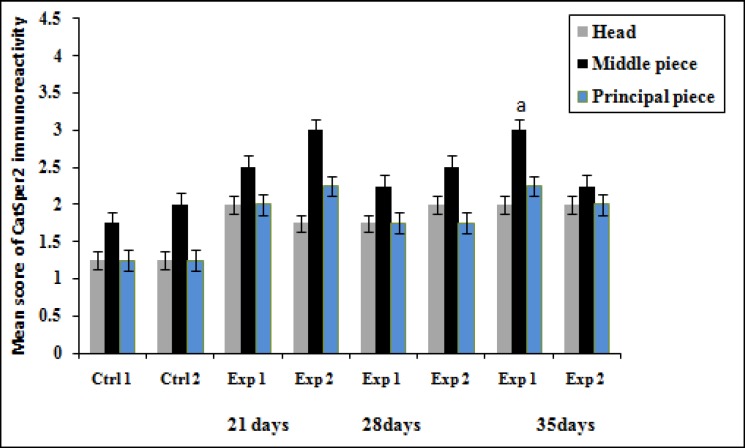
Effect of vitamin E treatment on protein levels of CatSper 2 in the aged and young mice Values expressed as means+SD, n=12 for each group. Exp: experimental, Ctrl: control

^a ^significant differences (p<0.05) between control 1 and experimental 1 group

Figure 3 shows the immunohistochemistry localization of CatSper protein and Figure 4 and Figure 5 demonstrate the estimate of immunoreactivity of CatSper 1 and CatSper 2 proteins in mouse sperm. Positive staining for CatSper proteins are shown in brown. Immunohistochemistry shows that CatSper 1 and CatSper 2 proteins are localized throughout the head and the flagellum (Figure 3 C-F). The intensity of CatSper staining in the mouse sperm from all groups was quantified. 

Staining immunoreactivity for CatSper1 was strong in the middle piece and weak in the head and the principal piece of sperm in the experimental group1 on the day 21. As vitamin treatment progressed from days 21 to days 28, CatSper1 reaction dramatically decreased in the middle piece of the sperm, but high level of staining was detected on the 35 day. The staining intensity increased in the middle piece of the sperm tail in the experimental group 2 on day 21 of vitamin administration. The amount of CatSper1 reaction increased gradually until day 28 of vitamin treatment, but low level of staining was recorded afterwards in the experimental group 2 (Figure 4). Moderate CatSper2 immunoreactivity was observed in the head and the tail of sperm on the days 21 and 28 whereas a strong signal was detected in the middle piece on the day 35 in the sperm cells of the experimental group1 (Figure 5). 

On the 21^st^ day of vitamin treatment, strong labeling was detected in the experimental group 2 in the middle piece of sperm tail but moderate in the head and principal piece of sperm tail. After this period, CatSper 2 reaction did not indicate remarkable changes. These findings were in agreement with their relative expression of CatSper genes. No staining was observed in control slides when PBS was used instead of primary antibody.

**Table I:** Effect of vitamin E treatment on sperm parameters in the aged and young mice

**Sperm Parameters **		**11-12 months old**		**2-3 months old**		**p-value** ^*^
		**Control group (1)**	**Experimental group (2)**	**Control group** ** (1)**	**Experimental group (2)**	
Day 21						
	Concentration (10^6^/mL)	3.80 ± 0.11^a^	4.18 ± 0.24^b^	4.40 ± 0.18^b^	4.76 ± 0.37^cd^	<0.001
	Motility (%)	42.00 ± 3.16^a^	51.25 ± 2.32^b^	77.75 ± 2.06^c^	86.50 ± 2.38^d^	<0.001
	Viability (%)	70.25 ± 4.50^a^	69.75 ± 0.50^a^	83.75 ± 6.40^a^	83.75 ± 4.35^a^	<0.001
	Normal morphology (%)	70.50 ± 3.32^a^	75.25 ± 7.14^ab^	81.00 ± 2.71^bc^	85.00 ± 3.74^c^	0.001
Day 28						
	Concentration (10^6^/mL)	3.87 ± 0.14^a^	4.10 ± 0.16^b^	4.35 ± 0.23^c^	4.92 ± 0.07^d^	<0.001
	Motility (%)	40.50 ± 4.12^a^	41.50 ± 4.20^a^	79.00 ± 2.94^c^	87.00 ± 1.82^d^	<0.001
	Viability (%)	71.50 ± 5.07^a^	70.25 ± 4.27^a^	82.75 ± 5.50^b^	82.75 ± 5.50^b^	<0.001
	Normal morphology (%)	70.00 ± 4.08^a^	74.25 ± 6.13^ab^	79.25 ± 0.96^bc^	86.00 ± 4.24^c^	<0.001
Day 35						
	Concentration (10^6^/mL)	3.75 ± 0.17^a^	4.11 ± 0.40^ab^	4.30 ± 0.29^b^	4.23 ± 0.93^b^	0.002
	Motility (%)	39.50 ± 0.58^a^	51.00 ± 2.44^b^	78.25 ± 3.95^c^	82.00 ± 2.44^c^	<0.001
	Viability (%)	71.75 ± 3.59^a^	69.50 ± 3.11^a^	84.00 ± 5.10^b^	84.00 ± 4.40^b^	<0.001
	Normal morphology (%)	72.25 ± 4.50^ab^	77.25 ± 1.71^bc^	78.00 ± 6.48^bc^	87.00 ± 1.82^d^	<0.001

Values are expressed as Mean±SD n=12 mice per group.

Data were analyzed by ANOVA test (p>0.05).

Different superscripts differed significantly within row at p<0.05 by Duncan post hoc test.

Values expressed as means+SD, n=12 for each group. Exp: experimental.

^a ^significant differences (p<0.05) between control and experimental group

^b^ significant differences (p<0.05) between experimental group 1 and experimental group 2 within one time-point

## Discussion

The results of the present study indicated that vitamin E treatment increases sperm parameters in the aged mice. Similarly, our previous study showed that administration of 0.2 mg/kg selenium improves sperm quality especially morphology and viability rates in the aged mice (23). Several mechanisms have been enunciated to explain the effects of aging process on sperm parameters (24). Epididymis plays an essential role in sperm motility. In epididymis, the site of sperm capacitation and maturation, aging process results in detrimental effects that this may explain how motility of spermatozoa might be affected by age. Sperm morphology is a sensitive index for the status of the germinal epithelium. 

Age-related changes may affect sperm morphology. Seminal vesicle secretes approximately sixty percents of the semen volume. A decrease in ejaculate volume could be caused by seminal vesicle inadequacy. Besides, smooth muscle atrophy in prostate may affect semen volume (24). There is a little data in the literature on the CatSper genes regulatory factors and the effects of antioxidant diet on the expression of CatSper genes. Previous studies have shown that gene expression affected by the antioxidants. Our results showed that administration of 106 mg/kg vitamin E could up-regulate CatSper 1 and CatSper 2 genes in the aged as well as young mice. 

However, administration of vitamin E in the aged mice caused more increase than that in the young mice. At the same time, a remarkable improvement was observed in sperm quality of the aged mice following vitamin E treatment, showing that there was a positive correlation between vitamin E treatment and improvement of sperm parameters. These results were consistent with our previous results that administration of 0.2 mg/kg selenium was significantly increased sperm parameters as well as CatSper genes expression (18). 

The relative intensity of CatSper genes expression in the aged mice treated with selenium was highly expressed on the days 21 and 28 but weakly expressed on the days 35 and 42 compared to those of the young mice. In the present study, a peak expression was observed in the aged treated with vitamin E on the day 35, whereas the highest level of CatSper genes expression was detected on the days 21 and 28, which were disagree with the results of our previous study. CatSper1 gene expression changes were more than that of the CatSper 2. In consistent with our results, among all four members of the CatSper genes family, more changes were observed in the expression of CatSper1.

It seems that CatSper1 has more powerful regulatory effects. Luo *et al* reported that CatSper1 mRNA was found to be down-regulated in a rat model of cyclophosphamide -induced oligoasthenozoospermia (25). However, administration of high-dose Yijingfang, a Chinese herbal decoction, increased CatSper1 expression in the epididymal sperm. Besides, sperm count and sperm motility improved following 35 days treatment with high dose of Yijingfang. Similar to this finding, we found that sperm parameters increased by up-regulating of CatSper1 and CatSper 2 expressions following dietary treatment with vitamin E. 

A study in Canada demonstrated supplementation with vitamin E affects the expression of glutathione S-transferases pi, 8, mu, and superoxide dismutase, all related to oxidative stress in the aged rats. Moreover, vitamin E deficiencies lead to increased expression of oxidative stress-related genes in the aging rat epididymis (26). In another study, Gan *et al* showed that intra peritoneal injection of 20 µg/kg selenium increased glutathione peroxidase (GSH-Px) expression in the testis and the liver (27). However, selenium treatment with 40 and 80 µg/kg predominantly decreased GSH-Px mRNA levels in the rat liver and the testis. This result was consistent with our study that an appropriate dose of the vitamin E, as an antioxidant, can increase genes expression in the testis. However, more researches are required to validate these findings.

Our immunocytochemistry studies showed the localization of CatSper1 and CatSper2 in the head, middle piece and principal piece of sperm tail. Previous studies have showed that CatSper proteins were found in both the middle piece and acrosomal region of sperm (7, 28). CatSper proteins were also localized to the principal piece of the sperm flagellum (7, 8, 28). According to their resembling domain structure, CatSper protein family was suggested to form a heteromeric channel in sperm but heterogeneous expression of CatSper genes family confirmed the hypothesis that CatSper 1 and CatSper 2 are likely responsible for the separate channels of CatSper 3 and CatSper 4 (3, 9). 

Our results also support this hypothesis. Staining intensity of CatSper1 and CatSper2 proteins was strong in the vitamin E treated mice; in particular, in the middle piece of flagellum. Interestingly, data obtained from CatSper1 and CatSper2 expression were consistent with their relative mRNA level. These findings showed that vitamin E treatment affected in both level of mRNA and protein. 

## Conclusion

The present study demonstrated that administration of 106 mg/kg Vitamin E could up-regulate CatSper1 and Catsper2 genes expression, which are two of the responsible genes for sperm mobility. Vitamin E treatment can improve sperm parameters, especially in terms of sperm concentration, motility and morphology rate; therefore, it seems that vitamin E treatment may be useful for treatment of aged subjects as well as infertile men with oligoasthenoteratospermia.
